# Body Mass Index in Mild Cognitive Impairment According to Age, Sex, Cognitive Intervention, and Hypertension and Risk of Progression to Alzheimer's Disease

**DOI:** 10.3389/fpsyt.2018.00142

**Published:** 2018-04-17

**Authors:** Soo Hyun Joo, Se Hee Yun, Dong Woo Kang, Chang Tae Hahn, Hyun Kook Lim, Chang Uk Lee

**Affiliations:** ^1^Department of Psychiatry, Seoul St. Mary's Hospital, College of Medicine, The Catholic University of Korea, Seoul, South Korea; ^2^Seocho Center for Dementia, Seoul, South Korea; ^3^Department of Psychiatry, Daejeon St. Mary's Hospital, College of Medicine, The Catholic University of Korea, Seoul, South Korea; ^4^Department of Psychiatry, Yeouido St. Mary's Hospital, College of Medicine, The Catholic University of Korea, Seoul, South Korea

**Keywords:** body mass index, mild cognitive impairment, Alzheimer's disease, intervention, CERAD

## Abstract

**Introduction:** Mild cognitive impairment (MCI) is a prodromal stage of dementia. The association of body mass index (BMI) and progression to Alzheimer's disease (AD) in MCI subjects according to age, sex, and cognitive intervention remains unknown. We investigated the relationship between BMI and the risk of progression to AD in subjects with MCI, as well as the effect of BMI on progression to AD depending on age, sex, cognitive intervention, and chronic diseases.

**Methods:** Three hundred and eighty-eight MCI subjects were followed for 36.3 ± 18.4 months, prospectively. They underwent neuropsychological testing more than twice during the follow-up period. The MCI subjects were categorized into underweight, normal weight, overweight, and obese subgroups. The associations between baseline BMI and progression to AD over the follow-up period were estimated using Cox proportional hazard regression models. Data were analyzed after stratification by age, sex, cognitive intervention, and chronic diseases.

**Results:** After adjustment for the covariates, the underweight MCI group had a higher risk of progression to AD [hazard ratio (HR): 2.38, 95% confidence interval (CI): 1.17–4.82] relative to the normal weight group. After stratifying by age, sex, cognitive intervention, and chronic diseases, this effect remained significant among females (HR: 3.15, 95% CI: 1.40–7.10), the older elderly ≥75 years old (HR: 3.52, 95% CI: 1.42–8.72), the non-intervention group (HR: 3.06, 95%CI: 1.18–7.91), and the hypertensive group (HR: 4.71, 95% CI: 1.17–18.99).

**Conclusion:** These data indicate that underweight could be a useful marker for identifying individuals at increased risk for AD in MCI subjects. This association is even stronger in females, older elderly subjects, the non-cognitive intervention group, and the hypertensive group.

## Introduction

Mild cognitive impairment (MCI) is a prodromal stage of dementia. According to epidemiological studies, approximately 5–15% of individuals with MCI will progress to dementia each year [1, 2]. Furthermore, there is no disease-modifying treatment for AD. Therefore, identifying risk factors at the MCI stage is critical because correcting modifiable risk factors can lower the incidence of dementia and the identification of non-modifiable risk factors can predict the progression of MCI to Alzheimer disease (AD).

Recently, there has been increasing evidence of the relationship between the body mass index (BMI) of normal cognitive individuals and risk of dementia. There are differences in the association between midlife BMI and dementia compared to late-life BMI and dementia. Being overweight or obese in mid-life is a risk factor for dementia [3–5], but in late-life being underweight is a risk factor [5–7]. However, there are few studies on the relationship between BMI and AD in MCI subjects. A recent study of 228 MCI subjects reported that the overweight or obese group had a reduced risk of both dementia and AD, while the underweight group had a higher risk of dementia but not AD, compared to the normal weight group [8]. Other study has investigated this relationship in MCI subjects, suggesting that, the underweight group had a higher risk while the obese group had a lower risk of AD compared to the normal weight group [9]. However, these two studies did not consider the heterogeneity of MCI subjects. The MCI group is composed of individuals with various demographics characteristics and life-styles. We suspected that BMI might have a different impact depending on individual's age, sex, cognitive intervention status, or chronic diseases, unlike finding from the two recent studies described above. In particular, cognitive intervention may improve cognitive reserve and prevent progression to dementia [10]. Therefore, the impact of BMI on the onset of AD may be different in individuals who have received cognitive intervention compared to a non-intervention group.

We hypothesized that BMI in MCI subjects can predict the progression to AD, and BMI may have a different effect depending on age, sex, cognitive intervention status, and chronic diseases. Therefore, we followed a cohort of MCI subjects prospectively to investigate the relationship between baseline BMI status and the risk of progression to AD in MCI subjects, as well as the effect of BMI on progression to AD depending on age, sex, cognitive intervention status, and chronic diseases.

## Materials and methods

### Study population

We recruited MCI subjects at the Seocho Center for Dementia, which is one of the 25 regional dementia support centers in Seoul, South Korea. Each participant underwent a detailed clinical interview and standardized neuropsychological battery, namely the Korean version of the Consortium to Establish a Registry for Alzheimer's disease (CERAD-K). Then, the psychiatrist employed a common standardized diagnostic assessment protocol that included measures for the diagnoses of normal cognition, MCI, and dementia. Participants diagnosed with MCI were eligible to take the CERAD-K test at least once every 6 months. Participants diagnosed with dementia were sent to the hospital for further evaluation. After diagnosis, participants could choose to attend cognitive intervention programs according to their particular cognitive level.

Enrollment started in September 2008 and ended in February 2015. The calculation of the follow-up period for each subject of the cohort was equal to the interval between enrollment and the diagnosis of dementia or end of follow-up. We screened 1,905 participants. We excluded 833 participants who were initially diagnosed with normal cognition or dementia. 684 participants were excluded because they did not follow-up. The final cohort consisted of 388 MCI participants who underwent neuropsychological testing more than twice. All participants were over 60 years old.

### Diagnostic assessments

A diagnosis of MCI was made by criteria recommended by the international working group [11]. Objective cognitive impairment of MCI was defined as a performance score of 1.5 *SD* below the respective age-, education-, and gender-specific normative means in at least one of the cognitive test included in the CERAD-K neuropsychological battery. This battery consisted of verbal fluency (VF), the 15-item Boston Naming Test (BNT), the Mini Mental Status Examination (MMSE), word list memory (WLM), constructional praxis (CP), word list recall (WLR), word list recognition (WLRc), and constructional recall (CR) [12]. All MCI subjects had an overall Clinical Dementia Rating of 0.5.

Dementia was defined as by the criteria of the fourth edition of the Diagnostic and Statistical Manual of Mental Disorders (DSM-IV) and required objective evidence of cognitive deficits (confirmed by neuropsychological testing) and social and/or occupational dysfunction (confirmed by impairments in ADL). We used the criteria of the National Institute of Neurological and Communicative Disorders and Stroke (NINCDS) and the Alzheimer's Disease and Related Disorders Association (ADRDA) for a diagnosis of AD [13].

### BMI categories

The weights and heights of all MCI subjects were measured using standard scales while the subjects were dressed in indoor clothing without shoes; using these data, BMI (kilograms/meters squared) was calculated at baseline. MCI subjects were categorized into four BMI subgroups based on the World Health Organization's (WHO) recommendations for Asian populations: underweight (BMI: <18.5 kg/m2), normal weight (18.5–22.9 kg/m2), overweight (23.0–24.9 kg/m2), and obese (BMI ≥ 25 kg/m2) [14–16].

### Cognitive intervention

Cognitive intervention is defined as engagement in a range of activities aimed at general enhancement of cognitive and social functioning in a nonspecific manner [10]. This term is a concept that includes cognitive training, cognitive rehabilitation, and cognitive stimulation. Our cognitive intervention includes group programs consisting of memory training, recreational activities (games, crafts, and learning to play musical instruments), and physical training implemented by the Seocho Center for Dementia. Each intervention was performed 2–3 times per week for 1 h. The presence of cognitive intervention was defined as attending one of these programs during the follow-up period.

### Statistical analyses

All analyses were performed with SPSS version 24 (SPSS Inc., Chicago, IL, USA). We compared demographic data using the Kruskal-Wallis test, or Chi-square test. The associations between BMI and progression to dementia over the follow-up period were estimated using Cox proportional hazard regression models and are shown as hazard ratios (HRs) with 95% confidence intervals (CIs). The multivariable models were as follows: First, we analyzed the associations without any adjustment. In Model 1, the independent effects of baseline BMI were evaluated after controlling for age, sex, and education. In Model 2, the CERAD-K total score, the presence of cognitive intervention, and the presence of each disease (hypertension, diabetes mellitus, hyperlipidemia, heart disease, or cerebrovascular disease) were added to Model 1. Finally, we divided the subjects into two groups by age (according to the median age), sex, the presence of cognitive intervention, and chronic diseases, separately. Statistical significance was defined as a *p*-value of 0.05. Values are presented as the mean ± standard deviation or frequency (percentage) as appropriate. Cumulative hazard curves according to BMI were derived from Model 2.

### Ethics statement

All participants voluntarily attended this study and gave written informed consent participate in the study. The Institutional Review Board of Catholic Medical Center approved this study protocol (KC17RESI0150).

## Results

### Baseline demographic characteristics

The baseline demographic characteristics of the 388 MCI subjects according to BMI category are summarized in Table 1. At baseline, 24 subjects were underweight, 160 normal weight, 120 overweight, and 84 obese. The mean age was 74.5 years and women were predominant (66.2%). The mean follow-up duration from the diagnosis of MCI was 36.3 months. 152 subjects attended cognitive intervention programs.

**Table 1:** Baseline clinical characteristics of MCI subjects according to BMI category (line No. 191).

	**All subjects**	**Underweight**	**Normal**	**Overweight**	**Obese**	***p*-value**
Number	388	24	160	120	84	
Female, *n* (%)	257 (66.2)	18 (75.0)	112 (70.0)	69 (57.5)	58 (69.1)	0.101
Baseline age, years	74.5 ± 7.6	77.1 ± 7.0	75.2 ± 8.3	74.4 ± 6.9	72.7 ± 6.9	0.029
<75, *n* (%)	200 (51.6)	7 (29.2)	76 (47.5)	66 (55.0)	51 (60.7)	0.026
≥75, *n* (%)	188 (48.5)	17 (70.8)	84 (52.5)	54 (45.0)	33 (39.3)	
Education, years	9.3 ± 5.0	10.4 ± 5.4	9.0 ± 4.8	9.4 ± 5.1	9.3 ± 5.1	0.445
Hypertension, *n* (%)	199 (51.7)	8 (33.3)	69 (44.0)	71 (59.2)	51 (60.7)	0.006
Diabetes mellitus, *n* (%)	70 (18.2)	1 (4.2)	29 (18.5)	26 (21.7)	14 (16.7)	0.232
Hyperlipidemia, *n* (%)	80 (20.8)	6 (25.0)	32 (20.4)	27 (22.5)	15 (17.9)	0.819
Heart disease, *n* (%)	46 (12.0)	3 (12.5)	13 (8.3)	14 (11.8)	16 (19.1)	0.110
Cerebrovascular disease, *n* (%)	28 (7.3)	1 (4.2)	10 (6.4)	8 (6.7)	9 (10.7)	0.557
Follow-up duration, months	36.3 ± 18.4	36.5 ± 22.1	37.1 ± 18.5	35.5 ± 18.7	36.0 ± 16.7	0.905
MMSE score	21.1 ± 4.1	20.4 ± 3.0	20.4 ± 4.4	21.8 ± 3.9	21.6 ± 3.8	0.007
CERAD-K total score	51.0 ± 14.1	48.0 ± 12.0	48.7 ± 15.3	52.6 ± 12.4	54.0 ± 13.8	0.014
Cognitive intervention						
Absent	236 (60.8)	19 (79.2)	106 (66.3)	58 (48.3)	53 (63.1)	0.004
Attended	152 (39.2)	5 (20.8)	54 (33.8)	62 (51.7)	31 (36.9)	

*Data are presented as the mean ± standard deviation or frequency (percentage). P-values were calculated by the Kruskal-Wallis test or chi square test. MCI, mild cognitive impairment; BMI, body mass index; MMSE, Mini-Mental Status Examination; CERAD-K, Korean version of the Consortium to Establish a Registry for Alzheimer's Disease*.

### Relationship between BMI and the progression to AD

**Figure 1:**
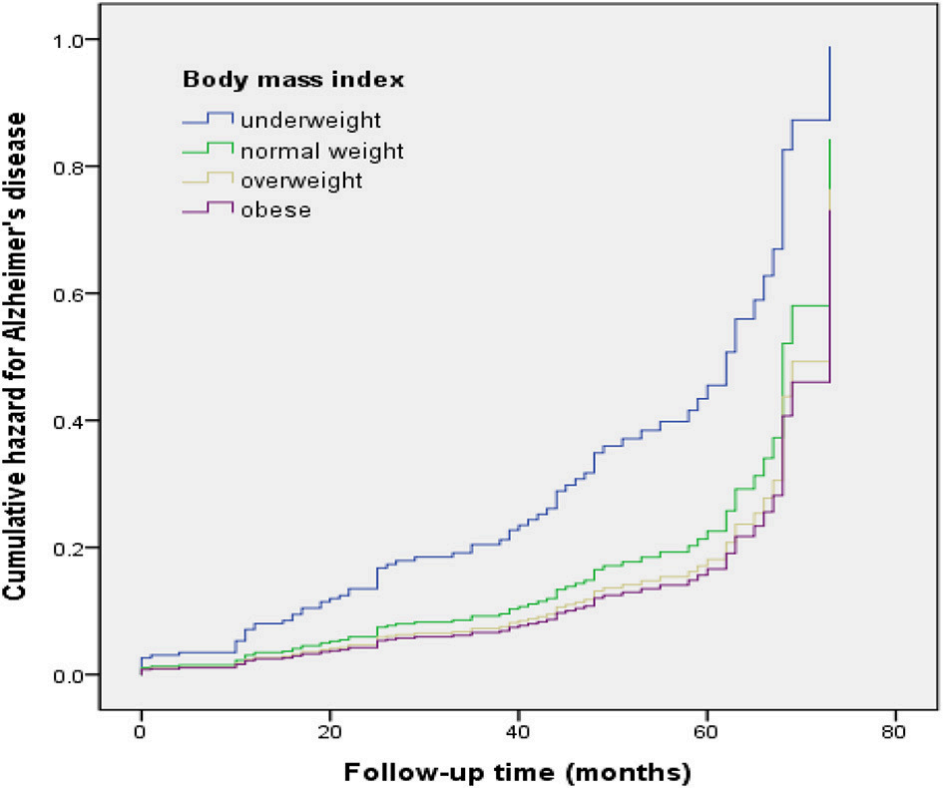
Cumulative risk curves of the effects of baseline body mass index categories on progression to Alzheimer's disease. The figure was derived from model 2 (adjusted for age, sex, education, CERAD-K total score, cognitive intervention, hypertension, diabetes mellitus, hyperlipidemia, heart disease, and cerebrovascular disease).

Of the MCI subjects, 12 of 24 underweight subjects (54.2%), 40 of 160 normal weight subjects (25.0%), 17 of 120 overweight subjects (14.2%), and 9 of 84 obese subjects (10.7%) progressed to AD. Prior to adjustment for covariates, the results of the Cox proportional hazard model showed that the underweight group had a higher risk of progression to AD (hazard ratio [HR]: 2.09, 95% confidence interval [CI]: 1.11–3.92) relative to the normal weight group. Adjusting for all covariates (Model 2) accentuated the risk of progression to AD (HR: 2.38, 95% CI: 1.17–4.82) (Table 2, Figure 1).

**Table 2:** Cox proportional hazard regression for risk of AD in MCI subjects according to baseline BMI (line No. 206).

	**Unadjusted**		**Model 1**		**Model 2**	
	**Crude HR (95% CI)**	***p-*value**	**Adjusted HR (95% CI)**	***p*-value**	**Adjusted HR (95% CI)**	***p*-value**
**BMI**						
Underweight	**2.09 (1.11–3.92)**	**0.023**	**2.00 (1.03–3.86)**	**0.039**	**2.38 (1.17–4.82)**	**0.017**
Normal weight	Reference		Reference		Reference	
Overweight	0.62 (0.35–1.10)	0.099	0.66 (0.37–1.17)	0.150	0.78 (0.42–1.45)	0.434
Obese	0.51 (0.25–1.06)	0.069	0.59 (0.28–1.23)	0.160	0.71 (0.33–1.53)	0.380

*Model 1: Adjusted for age, sex, and education; Model 2: Adjusted for Model 1 plus CERAD-K total score, cognitive intervention, hypertension, diabetes mellitus, hyperlipidemia, heart disease, and cerebrovascular disease. AD, Alzheimer's disease; MCI, mild cognitive impairment; BMI, body mass index; HR, hazard ratio; CI, confidence interval. Statistically significant values are shown in bold*.

### Effects of sex and age on the relationship between BMI and progression to AD

After stratifying by sex, underweight female had a higher risk of progression to AD (HR: 3.15, 95% CI: 1.40–7.10) than normal weight female in the fully adjusted model (Model 2). On the other hand, in male, there were no differences in the risk of progression to AD among the four BMI groups (Table 3).

**Table 3:** Cox proportional hazard regression for risk of AD in MCI subjects according to baseline BMI stratified by sex (line No. 225).

	**Unadjusted**		**Model 1**		**Model 2**	
	**Crude HR (95% CI)**	***p-*value**	**Adjusted HR (95% CI)**	***p*-value**	**Adjusted HR (95% CI)**	***p*-value**
Males			*n =* 129			
**BMI**						
Underweight	1.01 (0.21–4.80)	0.992	0.91 (0.19–4.40)	0.906	0.52 (0.09–2.99)	0.465
Normal weight	Reference		Reference		Reference	
Overweight	0.77 (0.30–1.99)	0.585	0.75 (0.28–2.02)	0.573	0.60 (0.20–1.86)	0.379
Obese	0.75 (0.20–2.79)	0.665	0.92 (0.24–3.53)	0.899	1.20 (0.26–5.55)	0.818
Females			*n =* 257			
**BMI**						
Underweight	**2.58 (1.29–5.16)**	**0.008**	**2.57 (1.23–5.38)**	**0.012**	**3.15 (1.40–7.10)**	**0.006**
Normal weight	Reference		Reference		Reference	
Overweight	0.53 (0.25–1.11)	0.094	0.57 (0.27–1.21)	0.145	0.82 (0.37–1.84)	0.632
Obese	0.44 (0.18–1.07)	0.070	0.51 (0.21–1.24)	0.135	0.64 (0.25–1.63)	0.351

*Model 1: Adjusted for age, sex, and education; Model 2: Adjusted for Model 1 plus CERAD-K total score, cognitive intervention, hypertension, diabetes mellitus, hyperlipidemia, heart disease, and cerebrovascular disease. AD, Alzheimer's disease; MCI, mild cognitive impairment; BMI, body mass index; HR, hazard ratio; CI, confidence interval. Statistically significant values are shown in bold*.

Age was divided into two groups according to the median age. Stratification of the full model by age revealed that the four BMI groups had no differences in the risk of progression to AD in younger elderly group (age < 75), while the underweight group had a higher risk of progression to AD (HR: 3.52, 95% CI: 1.42–8.72) than the normal weight group in older elderly group (age ≥ 75) (Table 4).

**Table 4:** Cox proportional hazard regression for risk of AD in MCI subjects according to baseline BMI stratified by age (<75, ≥75) (line No. 234).

	**Unadjusted**		**Model 1**		**Model 2**	
	**Crude HR (95% CI)**	***p-*value**	**Adjusted HR (95% CI)**	***p*-value**	**Adjusted HR (95% CI)**	***p*-value**
Age < 75			*n =* 198			
**BMI**						
Underweight	**3.29 (1.04–10.45)**	**0.043**	2.90 (0.88–9.62)	0.082	0.81 (0.18–3.73)	0.788
Normal weight	Reference		Reference		Reference	
Overweight	0.55 (0.19–1.55)	0.256	0.58 (0.20–1.65)	0.309	0.47 (0.15–1.48)	0.197
Obese	0.63 (0.20–1.99)	0.429	0.69 (0.22–2.19)	0.530	0.48 (0.14–1.63)	0.238
Age≥75			*n =* 183			
**BMI**						
Underweight	1.63 (0.76–3.49)	0.212	1.77 (0.80–3.94)	0.160	**3.52 (1.42–8.72)**	**0.007**
Normal weight	Reference		Reference		Reference	
Overweight	0.70 (0.35–1.40)	0.315	0.79 (0.39–1.59)	0.505	1.17 (0.53–2.58)	0.693
Obese	0.54 (0.21–1.40)	0.204	0.54 (0.21–1.42)	0.214	0.93 (0.34–2.56)	0.893

*Model 1: Adjusted for age, sex, education; Model 2: Adjusted for Model 1 plus CERAD-K total score, cognitive intervention, hypertension, diabetes mellitus, hyperlipidemia, heart disease, and cerebrovascular disease. AD, Alzheimer's disease; MCI, mild cognitive impairment; BMI, body mass index; HR, hazard ratio; CI, confidence interval. Statistically significant values are shown in bold*.

### Effects of cognitive intervention on the relationship between BMI and progression to AD

Stratification of the full model by cognitive intervention revealed the negative effect of underweight to be limited to the non-intervention group (Model 2, Table 5). In the cognitive intervention group, the underweight group had no risk of progression to AD while the overweight group had a lower risk of progression to AD (HR: 0.37, 95% CI: 0.14–0.99) than the normal weight group. On the other hand, in group without cognitive intervention underweight group had a higher risk of progression to AD (HR: 3.06, 95% CI: 1.18–7.91) than the normal weight group.

**Table 5:** Cox proportional hazard regression for risk of AD in MCI subjects according to baseline BMI stratified by cognitive intervention (line No. 251).

	**Unadjusted**		**Model 1**		**Model 2**	
	**Crude HR (95% CI)**	***p-*value**	**Adjusted HR (95% CI)**	***p*-value**	**Adjusted HR (95% CI)**	***p*-value**
Cognitive intervention-absent			*n =* 230			
**BMI**						
Underweight	**2.33 (1.07–5.09)**	**0.033**	**2.50 (1.09–5.69)**	**0.030**	**3.06 (1.18–7.91)**	**0.021**
Normal weight	Reference		Reference		Reference	
Overweight	0.89 (0.42–1.88)	0.7604	1.02 (0.47–2.18)	0.966	1.64 (0.71–3.78)	0.247
Obese	0.53 (0.20–1.39)	0.1946	0.62 (0.23–1.67)	0.347	0.87 (0.29–2.59)	0.803
Cognitive intervention-attended			*n =* 139			
**BMI**						
Underweight	1.91 (0.63–5.76)	0.250	1.74 (0.56–5.38)	0.338	3.59 (0.91–14.17)	0.068
Normal weight	Reference		Reference		Reference	
Overweight	**0.34 (0.14–0.82)**	**0.016**	**0.32 (0.13–0.80)**	**0.015**	**0.37 (0.14–0.99)**	**0.048**
Obese	0.47 (0.16–1.40)	0.172	0.50 (0.16–1.57)	0.237	0.51 (0.17–1.59)	0.249

*Model 1: Adjusted for age, sex, and education; Model 2: Adjusted for Model 1 plus CERAD-K total score, cognitive intervention, hypertension, diabetes mellitus, hyperlipidemia, heart disease, and cerebrovascular disease. AD, Alzheimer's disease; MCI, mild cognitive impairment; BMI, body mass index; HR, hazard ratio; CI, confidence interval. Statistically significant values are shown in bold*.

### Effects of chronic diseases on the relationship between BMI and progression to AD

After stratifying by hypertension, underweight hypertensive group had a higher risk of progression to AD (HR: 4.71, 95% CI: 1.17–18.99) than normal weight hypertensive group in the fully adjusted model (Model 2). On the other hand, there were no differences in the risk of progression to AD among the four BMI groups in group without hypertension (Table 6).

**Table 6:** Cox proportional hazard regression for risk of AD in MCI subjects according to hypertension (line No. 265).

	**Unadjusted**		**Model 1**		**Model 2**	
	**Crude HR (95% CI)**	***p-*value**	**Adjusted HR (95% CI)**	***p*-value**	**Adjusted HR (95% CI)**	***p*-value**
Hypertension-no			*n =* 184			
**BMI**						
Underweight	1.90 (0.88–4.10)	0.105	1.78 (0.81–3.94)	0.154	1.94 (0.78–4.83)	0.153
Normal weight	Reference		Reference		Reference	
Overweight	0.54 (0.24–1.21)	0.133	0.53 (0.24–1.18)	0.119	0.41 (0.17–1.00)	0.050
Obese	0.25 (0.06–1.06)	0.060	0.27 (0.06–1.15)	0.077	0.25 (0.06–1.07)	0.062
Hypertension-yes			*n =* 197			
**BMI**						
Underweight	2.88 (0.90–9.21)	0.074	2.91 (0.80–10.60)	0.106	**4.71 (1.17–18.99)**	**0.030**
Normal weight	Reference		Reference		Reference	
Overweight	1.01 (0.41–2.47)	0.990	1.27 (0.51–3.15)	0.605	1.86 (0.68–5.04)	0.224
Obese	1.00 (0.39–2.57)	0.993	1.25 (0.47–3.29)	0.657	1.38 (0.49–3.85)	0.544

*Model 1: Adjusted for age, sex, and education; Model 2: Adjusted for Model 1 plus CERAD-K total score, cognitive intervention, hypertension, diabetes mellitus, hyperlipidemia, heart disease, and cerebrovascular disease. AD, Alzheimer's disease; MCI, mild cognitive impairment; BMI, body mass index; HR, hazard ratio; CI, confidence interval. Statistically significant values are shown in bold*.

After stratifying by DM, underweight non-diabetic group had a higher risk of progression to AD (HR: 2.35, 95% CI: 1.12–4.95) than normal weight non-diabetic group in the fully adjusted model (Model 2). On the other hand, there were no differences in the risk of progression to AD among the four BMI groups in group with DM. However, statistical power was very low because the sample size of underweight subjects with DM was too small (*N* = 1). Other chronic diseases such as hyperlipidemia, heart disease, and cerebrovascular disease did not affect the relationship between underweight and progression to AD in MCI subjects.

## Discussion

This is the first prospective clinical study to confirm the relationship between BMI and AD considering the heterogeneity of MCI subjects. Our major findings were as follows: First, underweight MCI subjects had a higher risk of progression to AD relative to the normal weight group. Second, the negative effects of underweight on progression to AD were even stronger in females, older elderly, the non-cognitive intervention group and the hypertensive group.

Our first finding that underweight MCI subjects had a higher risk of progression to AD is in line with previous findings [8, 9, 17]. There are various explanations about the relationship between underweight and AD. Aging itself is associated with weight loss; however, weight loss may be accelerated before diagnosis of AD [18]. In other words, weight loss may be either a potential cause of dementia or it may be an early manifestation of an underlying dementia. Lower BMI may reflect a decrease in muscle mass or a decrease in fat. Some studies have shown that cognitive decline is associated with muscle loss [19, 20]. Poor nutritional status associated with reduced production of leptin, lack of vitamins, and essential fatty acids, can lead to oxidative damage to neuronal cells, and consequential acceleration of neurodegenerative processes [21]. Level of serum leptin, an adipocyte-derived peptide hormone have been lower in AD patients with BMI <20 compared to those with BMI >25 [22]. Another possible explanation is the degeneration of the mesial temporal cortex because limbic structures within the mesial temporal lobe are involved in appetite, feeding behaviors, memory, and emotional regulation all of which could potentially affected to body weight [23].

In our study, the association between underweight and progression to AD was restricted in females, and was not found in males. While these findings require further study and confirmation, one possible explanation for the observed gender difference may be hormonal factors. It is suggested that lower estrogen levels are potentially associated with AD in studies based on the association between estrogen replacement therapy in postmenopausal women [24, 25]. Because adipose tissue contributes to the production of estrogen, the circulating levels of estrogen are lower in women with less adipose tissue. Estrogens may affect cognition by binding to estrogen receptors that are located throughout the brain, especially in regions such as the hippocampus and amygdala which are involved in learning and memory [26].

We also found that age affected the relationship between underweight and progression to AD in MCI subjects. In the older elderly group, underweight predicted higher progression to AD, but not in the younger elderly group. In previous studies, there was a difference in the effect of BMI on the onset of dementia according to age. Obesity in midlife is associated with incident AD [3, 5], whereas underweight in the elderly is associated with AD [6, 7]. This finding highlights the importance of stratifying subjects by age in studies of BMI and dementia and suggests that the older elderly should be considered a separate population from the larger majority of elderly younger than 75.

Our other finding was that the impact of BMI on the progression to AD was different according to the presence or absence of cognitive intervention. The effect of cognitive intervention may be explained by neuroplasticity. Neuroplasticity is defined as the brain's ability to adapt to changes in the environment through modification, reorganization, and creation of neural connections [27]. Many cognitive intervention programs attempt to enhance neuroplasticity of the brain. Therefore, cognitive intervention has a beneficial effect on cognitive decline in MCI subjects [28–30]. In the present study, cognitive intervention did not affect the progression of MCI to AD. However, we found that cognitive intervention affects the relationship between BMI and the progression to AD in MCI subjects. Underweight still increased the risk of progression to AD in the non-intervention group, while overweight had a protective effect on the progression to AD in the intervention group. It may be an inaccurate interpretation that cognitive intervention contributes to reducing the impact of underweight on the progression to AD, therefore further studies on the impact of cognitive intervention are needed.

Finally, we also found that the association between underweight and progression to AD was stronger in hypertensive group, and was not found in non-hypertensive group. In the study by Sakakura et al. leanness in hypertensive elderly patients was associated with poor cognitive function [31]. The mechanism of cognitive decline in lean hypertensive patients is not clear. However, it is well known that hypertension affects cognitive decline in elderly subjects [32, 33]. There are evidences that white matter medullary arterioles are vulnerable to hypertension, it can cause microvascular dysfunction and narrowing, which can lead to cerebral hypoperfusion [34, 35]. Recent study found that hypertension was associated with worse cognitive function and hippocampal hypometabolism in AD patients [36]. Because underweight and hypertension are risk factors for AD, MCI patients with underweight and hypertension may be highly likely to progress to AD.

This study has several limitations. First, we did not specify MCI diagnosis by amnestic MCI, non-amnestic MCI, single domain MCI, or multi-domain MCI. Second, amyloid imaging, CSF analysis, or pathological studies were not performed to determine on AD diagnosis. Third, the APOE genotype may modify the association between BMI and cognitive decline; however, we could not investigate this association because more than half of the participants did not undergo APOE genotyping. Fourth, this study only analyzed the presence or absence of cognitive intervention without taking the type of cognitive intervention and the duration of attendance into consideration. Fifth, we performed stratification analysis based on chronic diseases which may affect BMI, but we could not found the potential effects of chronic diseases such as DM on MCI and the progression to AD because of the small sample size. Furthermore, we did not investigate the treatment of chronic diseases. Sixth, Muscle loss is also known to be related to cognitive decline, but we could not consider sarcopenic obesity because we didn't investigate muscle mass. Finally, we did not measure longitudinal changes of BMI over time. Nonetheless, this study has several strengths. This study not only identified an effect of underweight on the progression to AD in MCI, but was also the first study to analyze the effects of sex, age, cognitive intervention, and chronic diseases on the relationship between BMI and progression to AD in MCI subjects. Other strength of this study is that the CERAD-K, which is a more accurate cognitive function assessment tool than the MMSE, was used to diagnose MCI and AD. Also, the CERAD-K total score and cognitive intervention attendance, which may affect progression to AD in MCI, were used covariates in this study, which was not done in previous studies. Furthermore, BMI is a biomarker that can be measured easily in clinical practice, so this outcome will be useful to clinicians.

In conclusion, although it is not known whether underweight is the cause of AD or preclinical symptom, these data indicate that low baseline BMI could be a useful marker for identifying individuals at increased risk for AD in MCI subjects. This result is stronger in females, older elderly, the non-cognitive intervention group, and the hypertensive group. Therefore, age and sex specific BMI control might be needed for delaying progression to AD in MCI subjects, and individuals who do not receive cognitive intervention or who have hypertension might need more BMI control.

## Author contributions

CL planned the design and carried out the supervision of all parts of this study; SJ recruited participants, analyzed the data and drafted the manuscript; SY managed participants and collected the data; DK, CH, and HL collected the data and performed statistical analysis. All authors read and approved the final manuscript.

### Conflict of interest statement

The authors declare that the research was conducted in the absence of any commercial or financial relationships that could be construed as a potential conflict of interest.
